# Topographic Organization of Saccade-Related Response Field Properties in the Marmoset Posterior Parietal Cortex

**DOI:** 10.1523/ENEURO.0287-25.2025

**Published:** 2025-10-07

**Authors:** Joanita F. D’Souza, Jessima M. Rich, Shaun L. Cloherty, Nicholas S. C. Price, Maureen A. Hagan

**Affiliations:** ^1^Department of Physiology and Biomedicine Discovery Institute, Monash University, Clayton, Victoria 3800, Australia; ^2^School of Engineering, RMIT University, Melbourne, Victoria 3001, Australia

**Keywords:** electrophysiology, laminar, marmoset, parietal, saccades, topography

## Abstract

Despite various histological, electrophysiological, and imaging studies, the topographic organization of saccade-related activity in the posterior parietal cortex (PPC) has been notoriously difficult to characterize. In part, this is because areas of interest in PPC are often embedded deep in sulci in macaques and humans. Understanding the extent of topographic organization in PPC can provide insights into the computation contributions of PPC. The lissencephalic cortex of the common marmoset offers a unique opportunity to investigate fine-scale topographic organization in PPC. Recordings were obtained from the PPC of two male marmosets performing a visually guided center-out saccade task with 8 or 36 peripheral targets using multichannel electrode arrays with 100 μm spacing. By plotting the pattern of saccade direction tuning preferences across all penetrations and cortical depths, we uncovered topographic organizational features within the PPC. Like other primates, multiunits in marmoset PPC tend to prefer saccade targets in the contralateral visual field. The results detail how preference for saccadic direction changes in a systematic manner across cortical distance, such that response units closer in proximity tend to show systematic changes in their tuning preferences. Across cortical distance, the visual field was also systematically encoded but reversals in direction varied across penetrations. The analysis highlights the likelihood of multiple representations of the visual field for saccade direction preference across PPC. These novel findings suggest a possible functional organization of saccade-related activity in marmoset PPC, giving insights into the computational capacity of the PPC.

## Significance Statement

Topographic maps are found across the primate brain, particularly in visual areas. A fundamental feature of these maps is that neurons involved in similar computations are spatially clustered. Moving from early sensory areas to higher-order areas, the degree of topographic organization decreases. This may allow high-order areas more flexibility and to respond to stimuli in a dynamic environment. The extent to which the posterior parietal cortex (PPC) shows topographic organization has been a subject of debate. This study presents the first detailed mapping of saccade direction topography in marmoset monkeys. These results provide novel insights into the topographic organization of the PPC in primates and provide insights into its computational role in visual behavior.

## Introduction

Topographic maps are a ubiquitous organizational feature in the primate brain (Kaas, 1997), likely evolved to minimize connection distances between cells, reduce metabolic demands, and improve computational efficiency (Kohonen, 1982; Klyachko and Stevens, 2003; Yu et al., 2020). In particular, retinal topography—the systematic and continuous representation of the visual field—propagates through the visual cortex (Wandell et al., 2007). However, while strongest in early visual areas, retinotopic organization and lateralization decrease moving up the visual hierarchy (Felleman and Van Essen, 1991; Jack et al., 2007). This decreased topography may reflect a shift from local processing of visual space toward more global processing, allowing higher-order areas the flexibility to perform more complex computations across regions of visual space, like shifting spatial or feature attention.

The degree to which the posterior parietal cortex (PPC) is topographically organized has been a subject of ongoing debate. Electrophysiological recordings in macaques have yielded inconsistent results, from coarse contralateral representations (Blatt et al., 1990; Ben Hamed et al., 2001) to little evidence of retinotopy (Platt and Glimcher, 1998), reflecting both true variability and the challenge of accessing PPC deep within the intraparietal sulcus. Functional magnetic resonance imaging (fMRI) studies offer a broader view of visual field representation in PPC but also vary in their descriptions of topography, differing in the number, arrangement, and organization of maps across both humans and macaques (Schluppeck et al., 2006; Silver and Kastner, 2009; Patel et al., 2010; Arcaro et al., 2011). In humans, PPC maps are typically clustered with shared foveal representations and strong contralateral bias. Silver and Kastner (2009) identified up to seven adjacent maps, whereas Schluppeck et al. (2006) identified only three, possibly due to paradigm differences. In macaques, work by Patel et al. (2010) described a single contralateral map, but higher-resolution, wide-field mapping by Arcaro et al. (2011) revealed multiple adjacent maps with foveal and peripheral subdivisions. Discrepancies likely reflect both anatomical differences and methodological factors such as spatial resolution, stimulus extent, and task demands. Indeed, Patel et al. (2010) proposed that the organization of macaque lateral interparietal area (area LIP) may reflect the behavioral relevance of stimuli rather than passive visual input. Both macaque (Savaki et al., 2010) and human (Kagan et al., 2010) studies have shown that saccade targets elicit greater contralateral bias and topographic organization than visual stimuli alone, with anatomical evidence supporting saccade direction maps in PPC (Savaki et al., 2010). Clarifying the nature and extent of PPC's topographic organization—particularly in relation to visual and behaviorally relevant stimuli—may offer key insights into the functional roles of PPC in higher-order cognition (Felleman and Van Essen, 1991; Jack et al., 2007; Patel et al., 2010).

The lissencephalic cortex of the marmoset monkey offers a unique opportunity to systematically investigate topographic maps in a primate brain. Importantly, the entirety of the marmoset PPC lies on the cortical surface. While marmosets are increasingly being used to study visual behavior, the extent to which areas of the PPC are functionally homologous to other primates is still being established (D’Souza et al., 2021). Like other primates, marmoset PPC encodes planned saccades and is modulated by visual stimuli, eye position, saccade direction, and amplitude (Ghahremani et al., 2017; Ma et al., 2020). However, detailed mapping of the response fields of cells in the marmoset PPC has yet to be conducted.

This study provides the first evidence of the topographic layout of saccade direction preferences in marmoset PPC. Marmosets were trained to make visually guided saccades to 8 or 36 target locations while simultaneously recording from linear arrays with 32 electrodes, spaced 100 µm apart. Across electrode penetrations, maps of the visual field were reconstructed across the cortical surface and depth. As observed in other primates, a predominance of the contralateral visual field was observed in marmoset PPC. Adjacent multiunits in PPC also typically showed small, systematic changes in preferred saccade direction, whereas across larger distances, the representation of the visual field was not continuous and repeated in different regions. These findings suggest that marmoset PPC is structured to mediate both local interactions in visual space and more complex interactions across regions of the PPC.

## Materials and Methods

Two adult male marmoset monkeys (*Callithrix jacchus*) served as subjects in this study (M1, 6 years old, 440 g; M2, 4 years old, 490 g). The marmosets were captive-born and housed at Monash University with *ad libitum* water; a diet of commercial pellets supplemented with fruit, vegetables, and mealworms; and occasional food treats for positive reinforcement. They were housed individually or in family groups within a larger communal room, with access to enriched indoor (∼27°C, 40–60% humidity, 12 h light/dark cycle) and outdoor enclosures for up to 6 h/d, 5 d/week. All procedures were approved by the Monash Animal Research Platform Animal Ethics Committee and followed the Australian Code of practice for the care and use of animals for scientiﬁc purposes.

### Surgical procedure

Prior to training on the behavioral task, both marmosets were implanted with a titanium head-post to stabilize the head during experiments and a titanium cranial chamber over PPC (NeuroNexus; Pomberger and Hage, 2019). The marmosets were first injected with atropine (0.2 mg/kg, i.m.) and diazepam (2 mg/kg, i.m.). After 30 min, anesthesia was induced with alfaxalone (8 mg/kg, i.m.; Jurox). The marmoset was then placed in a stereotaxic frame and stabilized using earbars, which had been covered in local anesthetic (2% xylocaine jelly). After intubation, the head was further stabilized with a palate bar and eye bars. Anesthesia was maintained by gas isoflurane (0.5–3%) in oxygen. Eyes were protected during surgery with liquid paraffin eye ointment. Once stabilized, a midline incision was made, the scalp was reflected, and the temporalis muscles were separated to expose the skull. Up to six titanium screws (diameter 1.5 mm, length 4 mm) were inserted 1–1.5 mm into the skull. The exposed surface of the skull was coated with a thin layer of dental adhesive (Supabond, Parkell). A head-post, which would stabilize the animal's head during experiments, was then placed on one hemisphere, as close to the midline as possible, with a transparent dental acrylic (Ortho-Jet; Lang Dental Mfg.). The margin was cleaned, and the skin sealed with surgical adhesive (Vetbond; 3M) to the base of the headcap. Animals received oral antibiotics for 7 d (cefalexin monohydrate, 30 mg/kg) and analgesia for 5 d (meloxicam, 0.2 mg/kg). In a separate surgery for M1 but the same surgery for M2, a craniotomy (9 mm in diameter) was performed over PPC (stereotaxic coordinates, anterior–posterior 0; medial–lateral 6; Paxinos et al., 2012; Majka et al., 2020) in one hemisphere (M1, left; M2, right), leaving the dura intact. The cranial chamber was then placed over this region and secured in place on the skull with a dental adhesive (Supabond, Parkell) and dental acrylic.

### Behavioral training

One week postsurgery, the marmosets were acclimated to sitting in a custom-designed primate chair and head-posted. Visual stimuli were presented on a 24 in Viewpixx/3D LCD monitor (VPixx Technologies) with a resolution of 1,920 × 1,080 px (W × H) and a refresh rate of 100 Hz. The stimulus monitor was positioned at a fixed viewing distance of 48 cm in front of the marmoset. All visual stimuli were generated in MATLAB (MathWorks) using Neurostim (https://klabhub.github.io/neurostim/) and the Psychophysics Toolbox extension (Brainard, 1997). Eye position was tracked monocularly with a video-based Eyelink 1000 system (SR Research). Horizontal and vertical eye positions of the right eye were recorded at a 1 kHz sampling rate. At the start of each session, eye positions were calibrated by presenting marmoset faces (3.5° visual angle) at different locations in the visual field, up to 10° eccentricity. The marmosets were rewarded with sweetened liquids such as fruit juice (up to 12 ml in a session) at the end of every successfully completed trial (∼0.035 ml a trial, through a New Era syringe pump system).

### Center-out saccade task design

**Figure 1.**
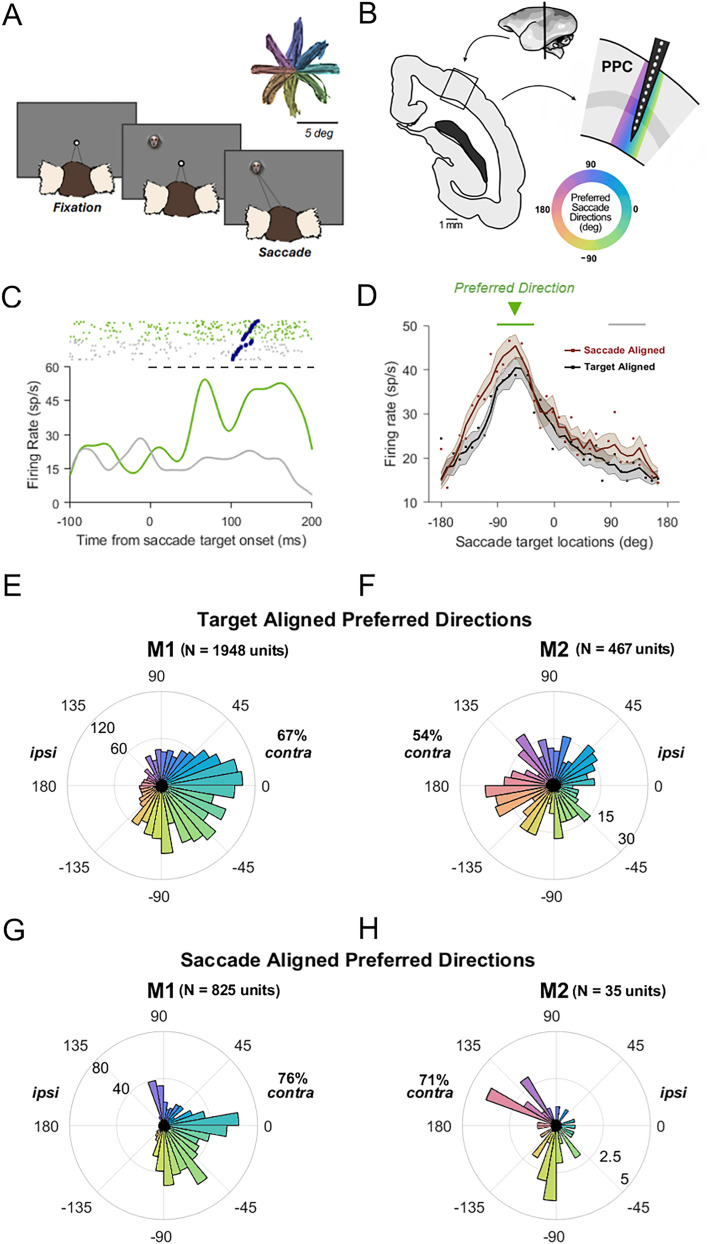
Behavioral task and experimental setup. ***A***, Center-out saccade task design. Marmosets were trained to fixate centrally before making a saccade to a peripheral target (a marmoset face) presented at 5° eccentricity to receive a reward. Peripheral saccade targets could appear at one of 8 or 36 equidistant locations. Inset, Eye movement traces from an example session with eight target locations (318 trials). ***B***, Implantation setup through a coronal section of the PPC. A 32-channel linear array was inserted to record activity from neurons across various cortical depths in PPC. The curved gray band marks the approximate location of Layer 4 (input layer), and the multicolored band illustrates how the array samples from adjacent cortical columns. If adjacent columns have adjacent preferred saccade directions, these preferences should systematically vary across recording positions for a tilted array insertion. ***C***, Example multiunit activity. The raster plot and peristimulus time histogram (PSTH) aligned to the saccade target onset, showing all trials within ±20° of the preferred saccade direction (green, −40°) and trials within ±20° of the opposite direction (gray). Rasters are sorted by saccadic reaction time, with blue squares indicating the saccade onset. The dashed horizontal line indicates the analysis window used for assessing target-aligned direction tuning: 0–200 ms after the target onset (black). ***D***, Saccade direction tuning curves for the same example unit shown in ***C***, computed in the target-aligned (black) and saccadic-aligned (brown; −50 to +200 ms around the saccade onset) analysis windows. Each point represents the mean firing rate for one of the 36 target locations. Bold lines indicate the ±10° moving average, and shaded areas represent the standard error of the mean. ***E–H***, Population tuning. Distributions of preferred saccade directions for M1 (***E***, ***G***) and M2 (***F***, ***H***), computed in the post-target–aligned window (***E***, ***F***, 1,948 and 467 units across 6 and 5 penetrations, respectively) and in the saccade-aligned analysis window (***G***, ***H***, 825 and 35 units across the same penetrations).

The center-out saccade task (Fig. 1*A*) required marmosets to first centrally fixate within a 2° radius window (700–900 ms for M1; 500–700 ms for M2) to initiate a trial. Thereafter, a conspecific face (2.5° visual angle) appeared at 5° eccentricity. On different days, targets could appear at one of either 8 or 36 equidistant locations. Marmosets had to saccade to this peripheral target within 500 ms [mean (SD) reaction time, M1, 124.2 (59.0) ms; M2, 159.5 (91.2) ms], and hold fixation (within a 2° radius window) for 200–400 ms to successfully complete a trial and receive a juice reward. Marmosets completed an average of 280 trials per 1–2 h session.

### Electrophysiological recordings

Once a marmoset was reliably performing the center-out saccade task to at least a 70% completion rate, a semichronic NeuroNexus p-drive housing a 32-electrode laminar probe (100 µm electrode spacing) was implanted through the recording chamber for up to 6 weeks. The structure of the semichronic microdrives allowed submillimeter changes in electrode position within the recording chamber, constrained by a radial grid (Pomberger and Hage, 2019). The external reference and ground wires were bridged and grounded to a silver wire that rested on the dural surface. Once the array had penetrated the dura and ground wire was in position, we filled the recording chamber with duragel (Cambridge NeuroTech).

In total, data were collected from six array penetrations in M1 (left hemisphere) and five penetrations in M2 (right hemisphere). Electrodes were mounted to a drive screw that allowed advancement of the electrode over the course of the implant as needed to isolate cells (each turn of the drive screw advanced the array 150–250 µm, 7–12 mm total drive range). Typically, on the day of insertion, the probe was advanced until neural activity was detected at depths of at least 400 µm in the brain. Thereafter, the array was slowly advanced 0–250 µm per day over the next 4 weeks until the array was fully implanted. This approach allowed sampling from all cortical depths in PPC.

Neural activity from each electrode was sampled at 30 kHz (OpenEphys). Multiunit spiking activity was extracted from each channel by high-pass filtering the raw voltage traces at 300 Hz applying a 5-standard-deviation threshold to identify putative spikes. Units with mean firing rates <5 spikes/s were excluded from further analyses. To extract local field potentials (LFPs), the raw voltage trace was low-pass filtered at 500 Hz and downsampled to 1 kHz.

### Determining cortical depth of recording electrodes

To measure the distance of each electrode channel from the cortical surface, the LFP for each channel was analyzed on the first and last trials of the center-out saccade task (−300 to 500 ms around the target onset) in each recording session. The shallowest electrode with a clearly defined LFP and a mean firing rate >5 spikes/s in the 200 ms following the target onset (target-aligned analysis window) was classified as the shallowest channel in the cortex. The distance of every subsequent electrode in the brain was then measured relative to the shallowest electrode recorded that day, as electrodes were spaced 100 µm apart. For recording sessions where the entire probe was already in the brain and the probe was still advanced further, the distance from the cortical surface was determined based on the number of turns of the drive screw. Because the insertion trajectory of the probe was not perfectly orthogonal to the cortical surface (Fig. 1*B*), these measurements of “cortical depth” also include some lateral displacement across the cortical surface.

### Calculating preferred saccade direction

All analyses were performed in MATLAB (MathWorks) using custom code and compatible toolboxes such as the circular statistics toolbox (Berens, 2009). To define the preferred saccade direction for each multiunit, the firing rate on each trial was calculated in the first 200 ms after the target onset (target-aligned analysis window; Fig. 1*C*). On days with 36 target locations, targets were grouped into eight equally spaced bins. The preferred saccade direction was defined as the angle of the resultant vector using the firing rates across all target bins. Multiunits were considered significantly tuned to saccade direction if the firing rate distribution across targets was nonuniform (*p* < 0.05; Rayleigh's test of nonuniformity; Fig. 1*D*). To test whether responses were more driven by the saccade itself, we repeated the tuning analysis using a second time window aligned to the saccade (saccade-aligned analysis window; −50 to +200 ms around the saccade onset). Multiunits that were not significantly tuned to saccade direction were excluded from further analysis.

### Quantifying visual topography

To test whether changes in preferred saccade direction across cortical distance were consistent with visual topography, two statistical tests were performed. First, a permutation test was performed to calculate the distribution of changes in preferred direction that would be expected by chance if distance between pairs of multiunits is not a factor. This was then compared with the median change in preferred direction across cortical distance (100–500 µm). Second, the correlation between changes in preferred direction and distance was calculated using Spearman's rank correlation.

To test whether preferred directions changed consistently over cortical distances, the sequence length (µm) and sequence visual angle (dov) was measured by many stepwise changes in preferred direction occurring before a reversal in direction. To minimize noise in this calculation, the mean preferred directions over a 200 µm moving window was then calculated for each electrode penetration.

### Histology

M1 was anesthetized with Alfaxan (8 mg/kg, i.m.; Jurox) and subsequently killed via an overdose of sodium pentobarbitone (100 mg/kg, i.v.). Following cardiac arrest, transcardial perfusion was performed using 0.1 M heparinized phosphate buffer (PB), pH 7.2, followed by 4% paraformaldehyde (PFA) in 0.1 M PB. The brain was postfixed in the same solution for 24 h and then cryoprotected by immersion in a series of 4% PFA-buffered sucrose solutions of increasing concentration (10, 20, and 30%). The brain was coronally sectioned at 40 μm. Myelin- and Nissl-stained series of sections were used to delineate boundaries for area LIP (see Majka et al., 2020, for details). Digitized sections from the left hemisphere of M1 were manually traced in Procreate (Apple, iPadOS app) to delineate key anatomical landmarks, including the cortical surface, lateral ventricles, and Layer 4. At the time of this publication, no histological analysis has been performed for M2, as the animal remains in experimental use.

## Results

Extracellular electrophysiological recordings were obtained from 11 penetrations in the PPC in two marmosets (six from M1 in the left hemisphere; five from M2 in the right hemisphere), while they performed a center-out saccade task (Fig. 1*A*). Marmosets were trained to fixate at a central target for a variable delay period. Following successful fixation, a marmoset face appeared at one of 8 or 36 locations at 5° eccentricity, which cued the animal to make an immediate saccade to the target. Neural activity was recorded using 32-channel probes with 100 µm spacing, allowing sampling of multiunits at distances of up to 3.5 mm apart. Although the probes spanned all cortical depths, they were not inserted orthogonal to the cortical surface and likely sampled across cortical columns, due to the curvature of the cortex (Fig. 1*B*). It was hypothesized that if preferred saccade direction were retinotopically organized, changes in preferred direction would be seen systematically along the length of the electrode. Alternatively, in the absence of topographic organization, preferred saccade direction should vary randomly between adjacent electrodes.

Consistent with findings in other primates (Blatt et al., 1990; Barash et al., 1991), many multiunits in the marmoset PPC exhibited robust tuning for saccade direction. Firing rates increased following the target onset for stimuli that evoked saccades in a unit's preferred direction but not for targets in the antipreferred direction (Fig. 1*C*, example unit). Firing rates also varied systematically for intermediate directions between the preferred and antipreferred targets (Fig. 1*D*, same unit). Across all electrodes and penetrations, 1,948 multiunits that were significantly tuned for saccade direction were identified in M1 and 467 units in M2 (Rayleigh's test for nonuniformity, *p* < 0.05 for M1 and M2). The majority of these units preferred targets in the visual field contralateral to the recording hemisphere (Fig. 1*E*, M1, 67%; 1,305/1,948 units; Fig. 1*F*, M2, 54%; 252/467 units), consistent with previous reports in macaques (Patel et al., 2010; Savaki et al., 2010; Arcaro et al., 2011).

Some units, like the example unit shown, displayed peaks in activity following both target presentation and the saccade. Because this task did not contain a delay period between target presentation and saccade, it is difficult to ascertain how much of the observed activity is driven by the visual target, saccade preparation, or the saccade itself. To test whether PPC units were more driven by postsaccade activity, we repeated the tuning analysis using a saccade-aligned window. Fewer units were found to have significant tuning using this window (Fig. 1*G*, M1, 825 units; Fig. 1*H*, M2, 35 units). Among multiunits significantly tuned in the saccade-aligned window, a similar contralateral bias was observed (Fig. 1*G*, M1, 76%, 627/825 units; Fig. 1*H*, M2, 71%, 25/35 units). These units were largely a subset of the units that showed tuning in the target-aligned window (M1, 88.4%, 729/825 units; M2, 91.4%, 32/35 units). This indicates that the majority of units in this dataset were tuned in a time window that included the visual onset of the target and the preparation for the saccade rather than in response to the saccade alone. For this reason, all subsequent analyses were conducted using the target-aligned window.

### Preferred saccade direction changes systematically in PPC

**Figure 2.**
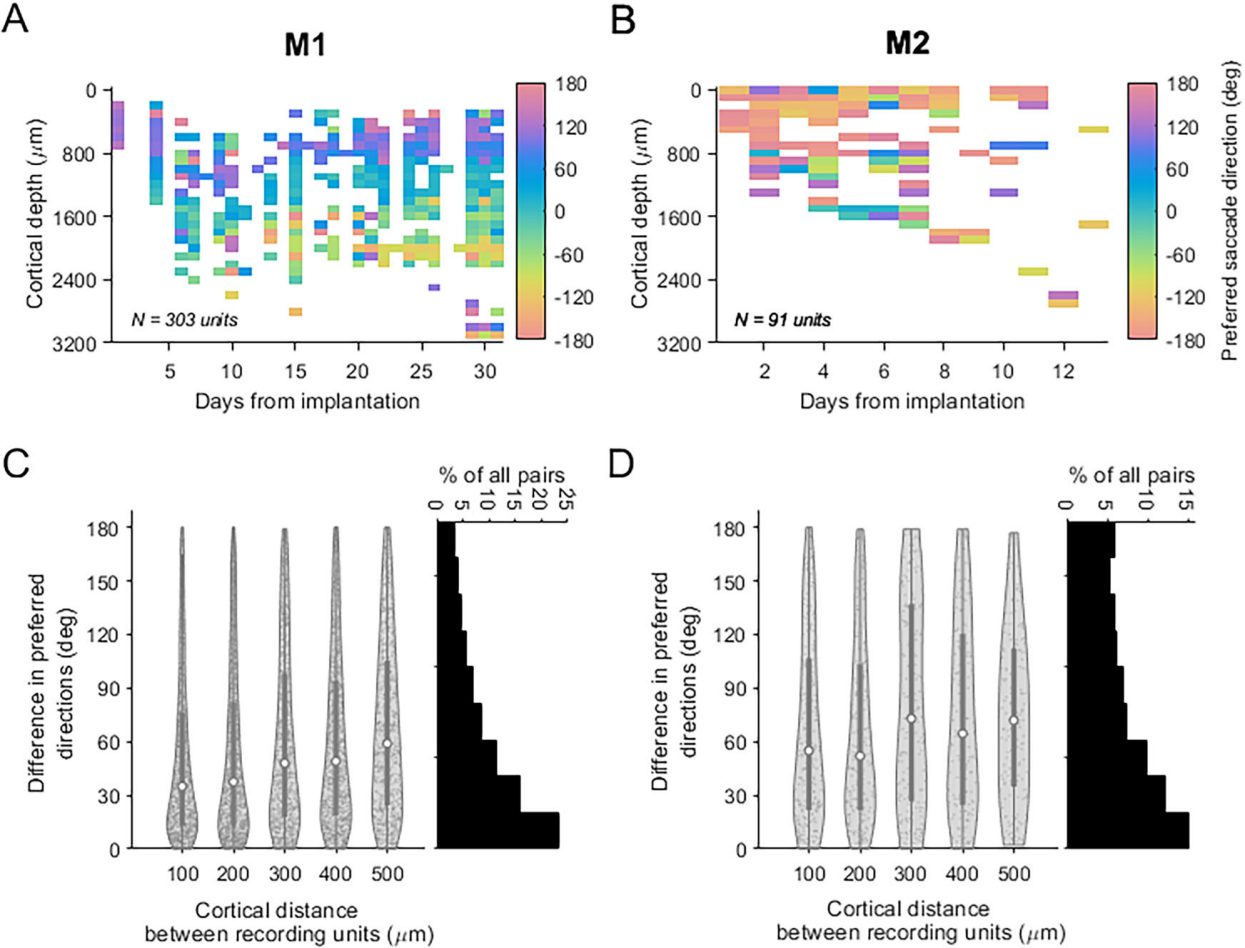
Saccade-related direction tuning systematically varied across cortical depth in PPC. Preferred saccade directions across cortical depth from example penetrations lasting 31 d in M1 (***A***) and 13 d in M2 (***B***). The color indicates preferred saccade direction. The violin plot distributions of the differences in preferred saccade directions for pairs of recorded multiunits separated by 100–500 µm of cortical distance, across all recording days in M1 (***C***) and M2 (***D***). Plots indicate the median differences in preferred direction (open circle) at each binned cortical distance from 100 to 500 µm, the boundaries of the first and third quartile (bolded line), and the total range of preferred direction differences. The frequency histograms (right insets in ***C*** and ***D***) depict the percentage of all pairs of recorded multiunits (M1, 5,698 pairs total; M2, 1,067 pairs total), across all preferred direction differences.

During electrode advancement over multiple days, a systematic shift in preferred saccade direction across the electrode length was observed in both M1 (Fig. 2*A*, example penetration) and M2 (Fig. 2*B*, example penetration). Due to the curvature of the marmoset cortex, probe insertions were not orthogonal to the cortical surface. Therefore, these changes likely reflect tuning differences across adjacent columns rather than within a single column (Fig. 1*B*). To test whether these variations in neuronal tuning were consistent with an organized topography, we analyzed the relationship between differences in preferred saccade direction and the physical distance between electrode contacts recording tuned multiunit activity. The analysis focused on multiunit pairs separated by 100–500 µm, as few pairs had larger separations.

Analyses revealed that smaller physical separations between pairs were associated with smaller differences in preferred direction. As the cortical distance between pairs increased, the differences in preferred direction generally became larger (M1, *R* = 0.14; *p* < 0.0001; M2, *R* = 0.08; *p* = 0.01; Spearman's rank correlation). The size of changes in preferred direction across distance was also tested to determine whether it was smaller than expected under a random, nontopographic organization. A permutation test was performed for each animal using the distribution of preferred directions. Changes in preferred direction at each cortical distance were found to be significantly smaller than predicted by chance. In M1 (Fig. 2*C*), the median difference in preferred direction for pairs separated by 100 µm was 35° (*p* < 1 × 10^−4^; permutation test), with differences generally increasing with separation distance, 37.5° at 200 µm (*p* < 1 × 10^−4^), 48° at 300 µm (*p* < 1 × 10^−4^), 49° at 400 µm (*p* < 1 × 10^−4^), and 59° at 500 µm (*p* < 1 × 10^−4^). Similarly, in M2 (Fig. 2*D*), the median difference at 100 µm was 55° (*p* < 1 × 10^−4^; permutation test), and it increased with separation distance, 52° at 200 µm (*p* < 1 × 10^−4^), 73° at 300 µm (*p* < 1 × 10^−2^), 64.5° at 400 µm (*p* < 1 × 10^−4^), and 72° at 500 µm (*p* < 1 × 10^−2^).

**Figure 3.**
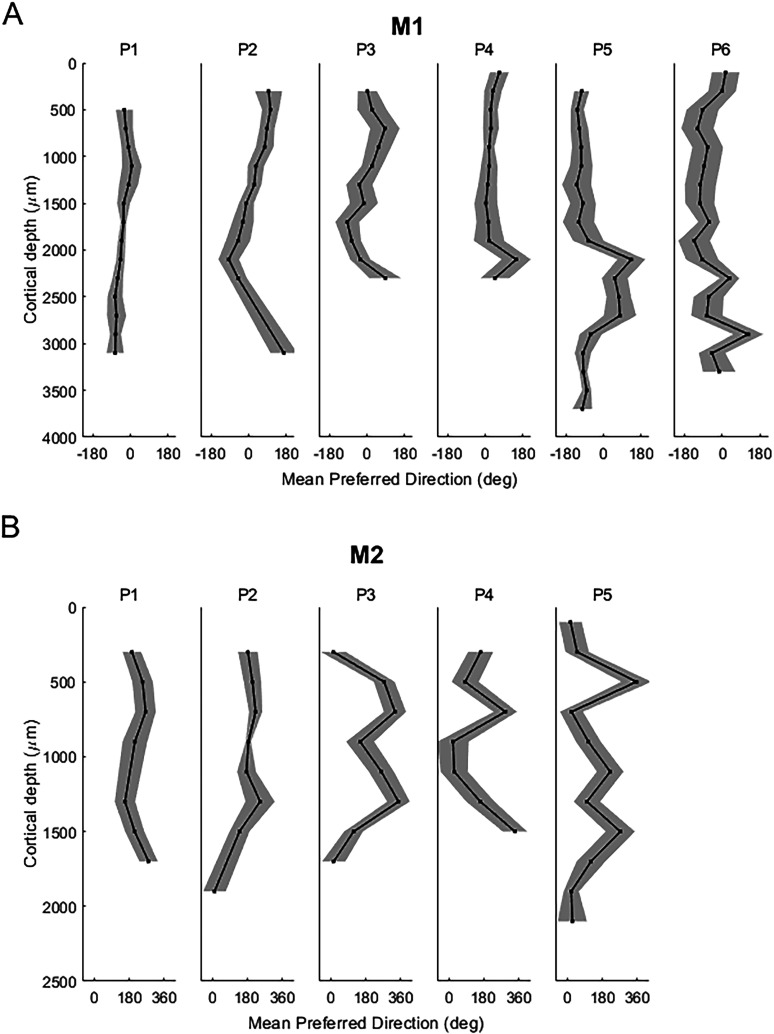
Preferred saccade direction for each penetration, time-averaged and smoothed over 200 µm. For each penetration, the preferred saccade direction was computed as a time-averaged measure and then spatially smoothed over a 200 µm window, for all penetrations in M1 (***A***) and M2 (***B***). The shaded region shows the temporal standard deviation. For each animal, the *x*-axis is centered at the preferred saccade direction along the horizontal meridian of the contralateral visual field (0 for M1, 180 for M2).

Small changes in preferred direction across nearby electrodes do not necessarily indicate that the visual field is systematically represented across the cortex. Next, we tested whether the changes in preferred saccade direction over distance moved consistently across the visual field or exhibited reversals. To quantify how preferred saccade direction changes across cortical distance, we calculated the average preferred saccade direction for all units within 200 µm bins and across all days within a penetration for each penetration (Fig. 3*A*, M1; Fig. 3*B*, M2). Generally, there was minimal variation in preferred saccade direction at each cortical depth, as is evident from the standard deviation error bar (mean, 30°).

A trend in changes in preferred saccade directions was ascertained. In some electrode penetrations, a systematic, consistent progression of preferred saccade direction across cortical depth was observed (e.g., Fig. 3*A*, P1 in M1; Fig. 3*B*, P2 in M2). In other penetrations, however, the progression of estimated preferred saccade direction moved back and forth across the visual field (e.g., Fig. 3*A*, P3 and P5 in M1; Fig. 3*B*, P3 and P5 in M2). Therefore, while nearby electrodes tend to represent changes in the visual field systematically, across larger cortical depths distances, the consistency in changes in preferred direction across the visual field varied across electrode penetrations.

To quantify the extent to which the visual field was represented before a reversal in direction, we calculated the length of sequences of changing preferred directions in a particular direction until a reversal of direction occurred. Specifically, for each electrode penetration (Fig. 3), the distance along the length of the electrode over which sequences of preferred directions advanced in one direction before reversing was measured. The extent of the visual field (in degrees of the visual angle) traversed for each sequence was also measured. It was hypothesized that longer sequences in one direction should correspond to a larger representation of the visual field. Longer sequences in one direction were found to correlate with larger portions of the visual field being represented in both monkeys (M1, *R* = 0.72; *p* = 8.7 × 10^−9^; *N* = 47 sequences; M2, *R* = 0.51; *p* = 8.4 × 10^−4^; *N* = 26 sequences; Spearman's rank correlation). For M1, the average sequence length was 344.7 ± 47.8 µm (mean ± SE) covering an average of 51.7 ± 0.2° of the visual angle. For M2, the average sequence length was 292.3 ± 43.3 µm covering an average of 71.2 ± 0.3° of the visual angle. It is important to note that the ability to sample sequence lengths is limited by the size and position of electrodes. Therefore, these measurements reflect the minimum rather than the maximum representations of the visual field. Nonetheless, the reversals in direction observed across many penetrations suggest multiple representations of the visual field may exist with marmoset PPC.

### Multiple representations of visual space across PPC

**Figure 4.**
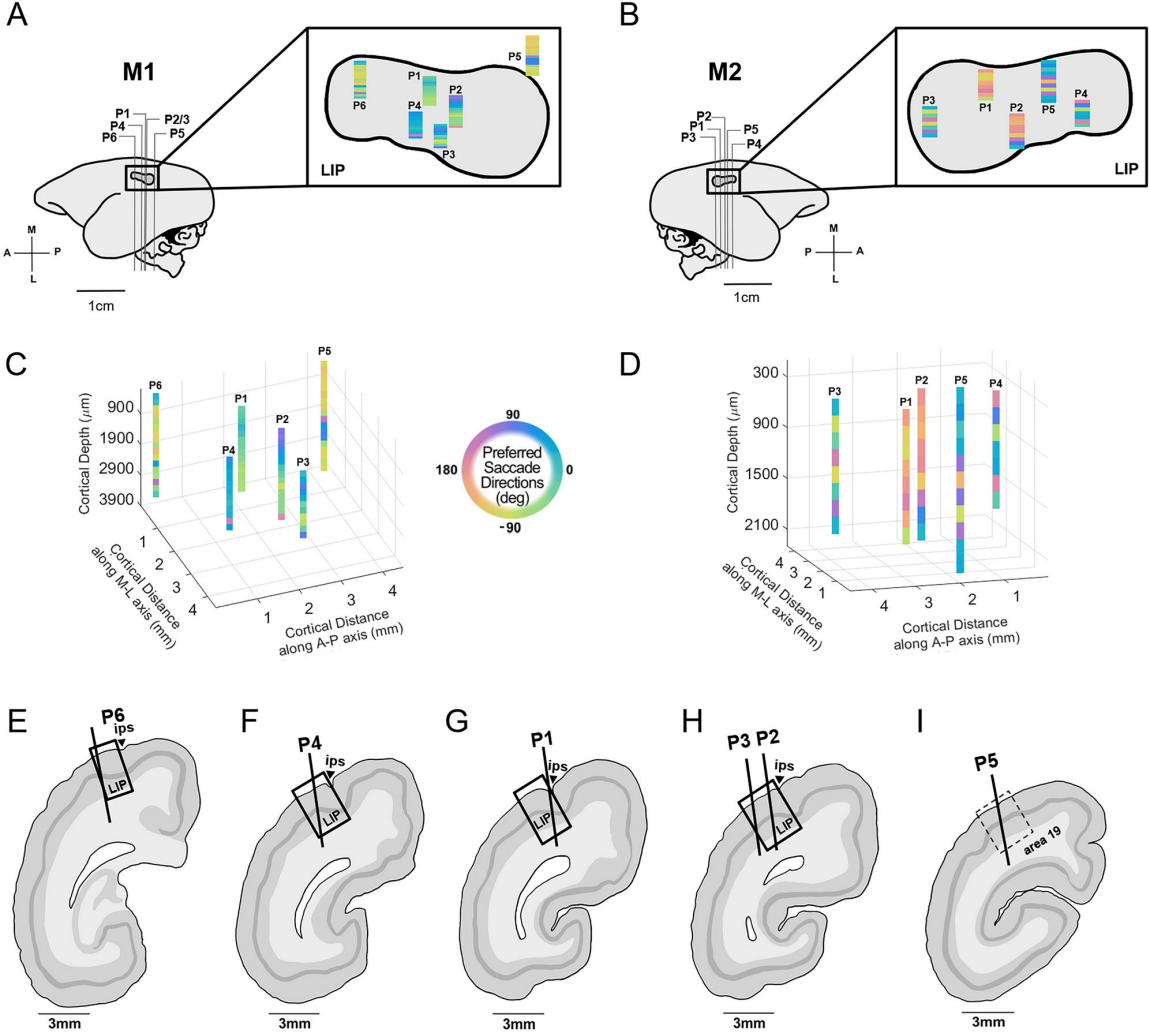
Multiple visual field representations revealed through the spatial organization of saccade direction preferences across array penetrations in area LIP. The approximate locations of all array penetrations (±0.5 mm) in M1 (***A***) and M2 (***B***) are shown across the surface of area LIP, relative to the boundaries of the implanted recording chambers (M1, left hemisphere; M2, right hemisphere). Chambers were stereotaxically positioned over area LIP (anterior–posterior 0, medial–lateral 6). Insets in panels ***A*** and ***B*** show the spatial distribution of array penetrations (6 in M1; 5 in M2) across the cortical surface of LIP, along with a coarse topographic map of saccade direction preference. Note, surface penetration sizes are illustrative only and not to scale. Panels ***C*** and ***D*** show the 3D topographic maps of preferred saccade direction across cortical depth for each penetration in M1 and M2, respectively. Colors represent time-averaged saccade direction preferences, smoothed over 200 µm. Given that precise penetration angles were not measurable, cortical depth is expressed as the distance along the array from the cortical entry point. Measured distances along the anterior–posterior (A–P) and medial–lateral (M–L) axis are relative to the center of the chamber. Panels ***E–I*** depict sketches of Nissl-stained coronal sections from M1 penetrations, ordered from anterior to posterior. Insertion angles were estimated based on chamber orientation. Layer 4 is shaded in dark gray, and anatomical landmarks—including area LIP, the intraparietal sulcus (IPS), and Area 19—are labeled according to the marmoset brain atlas (Paxinos et al., 2012; Majka et al., 2020).

Previous work in humans (Schluppeck et al., 2005, 2006; Silver et al., 2005) and macaques (Bakola et al., 2006; Arcaro et al., 2011) suggests that multiple representations of the visual field exist within the PPC. To test whether the reversals in preferred direction observed within penetrations reflected a single or multiple visual field representations, each array penetration was aligned based on its entry point relative to the cortical surface and mapped this to stereotaxic coordinates of area LIP, using the marmoset brain atlas (Paxinos et al., 2012; Majka et al., 2020; Fig. 4). To localize array penetrations, the spatial position of each site was estimated in M1 (Fig. 4*A*) and M2 (Fig. 4*B*) along the anterior–posterior axis (±0.5 mm; see Materials and Methods). Similar regions of visual space were represented at distinct cortical locations within the PPC. For example, rightward saccade preferences (∼0°) appeared at several penetrations in M1 (Fig. 4*A*, P1–P3), whereas leftward preferences (∼180°) were found at separate sites in M2 (Fig. 4*B*, P1, P2, P5). Reconstructing these penetrations in 3D (M1, Fig. 4*C*; M2, Fig. 4*D*) further illustrated how preferred saccade directions are distributed across the cortical surface, revealing repeated representations of the visual field. For one animal (M1), electrode locations were mapped onto postmortem Nissl-stained coronal sections (Fig. 4*E*–*I*). This confirmed that five out of six of the penetrations were located within the anatomically defined borders of area LIP (Paxinos et al., 2012; Majka et al., 2020). While the number of penetrations was insufficient to fully map the topography of the PPC, our results suggest that visual field representations are repeated at least three times across its surface—consistent with findings in macaques and humans (Schluppeck et al., 2006; Silver and Kastner, 2009; Arcaro et al., 2011).

## Discussion

To our knowledge, this is the first detailed evidence of fine-scale topographic organization of saccade direction preferences in the marmoset PPC. While previous work in macaques has demonstrated topographic representations of saccades in parietal areas using metabolic and fMRI measures (Kagan et al., 2010; Savaki et al., 2010), the results in this study extend these findings by revealing systematic changes in saccade direction preference at the level of adjacent multiunit recordings. It was found that nearby recording sites in PPC had more similar preferred directions than those farther apart and that changes in preferred direction were consistent across cortical distance. This suggested a fine-grained topographical organization that is consistent with a depth-dependent organization of visual space. However, reversals were also observed in the direction of visual field representation. Furthermore, saccades directed toward similar regions of visual space were represented at multiple, spatially distinct PPC sites. Together, these results are consistent with the presence of multiple visual field maps within the PPC. These findings advance our understanding of the fine-grained functional architecture of PPC and its computational role in visual behavior.

Topographic maps may emerge from efficient connectivity constraints. Spatial clustering of neurons with similar computations reduces the number of long-range connections across the brain, thereby reducing metabolic demands, reducing processing latencies, and facilitating synaptic plasticity (Kohonen, 1982; Klyachko and Stevens, 2003; Yu et al., 2020). Retinotopy in the visual system reflects this type of efficient and coherent organization of spatial information and is conserved throughout subcortical and cortical areas in the visual system (Wandell et al., 2007). However, moving from early visual areas into higher-order visual areas, topographic organization and retinotopic maps in particular become notably more coarse and inhomogeneous (Felleman and Van Essen, 1991; Jack et al., 2007). Recent computational modeling suggests that the decline in topographic precision across the visual hierarchy may result from activity-driven development, where secondary areas like PPC self-organize based on input from primary areas such as V1, under synaptic resource constraints (Imam and Finlay, 2020). Given the density of feedback projections and often widespread interconnectedness of networks involving the PPC, the decreased topography observed in higher-order areas might arise here due to constraints enforced by the asynchronous development with other more mature areas (Bourne and Rosa, 2006; Mundinano et al., 2015).

Previously, investigations into the strength of retinotopic maps in macaque PPC have yielded inconsistent conclusions. While some have reported clear delineations of the visual field between ventral and dorsomedial parts of PPC (Ben Hamed et al., 2001; Bakola et al., 2006; Patel et al., 2010), others have described a much coarser retinal topography (Blatt et al., 1990). Here, the lissencephalic structure of the marmoset brain permitted systematic sampling of cells at 100 µm spacing in a way that was not previously possible in other animal models. As a result, fine-scale topographic organization in PPC was demonstrated. The closer two neural multiunits were spatially, the smaller the difference in their preferred directions. Particularly, units within 100–200 µm shared very similar saccade direction preferences. Because our analysis used multiunit activity, it is possible that at 100 µm spacing, there is some contamination across multiunits resulting in artificially close estimates of preferred direction. However, even at 100 µm, a shift in preferred direction was generally observed (median shift in preferred direction was 35° for M1 and 55° for M2). Furthermore, the consistent and increasing shifts across cortical distance suggest multiunit contamination alone cannot explain these results. Some of these systematic changes in preferred saccade direction across the length of our electrode (see example penetrations in Fig. 2*A*,*B*) may be attributed to the curvature of the marmoset brain and the likely oblique angle of insertion of the recording probes. This may explain how units could be recorded across distances of up to 3 mm (Fig. 3*A*) even though the marmoset cortex in the PPC is estimated to be ∼2 mm thick (Atapour et al., 2019). Consequently, the pattern of changing saccade preferences across depth may be consistent with the idea that our electrodes are traversing multiple cortical columns with differing saccade direction preferences.

Over short distances, systematic and monotonic changes in neurons’ preferred saccade directions were observed. Over larger cortical distances, reversals in the direction of these monotonic changes were found. These reversals may explain why previous electrophysiology studies, with cells sampled at greater distances, reported “jumps” in the visual topography in the PPC (Blatt et al., 1990; Ben Hamed et al., 2001). It is also possible that fractured retinotopic maps are a by-product of a smaller brain size to body ratio. Partial coverage of the visual field, even in early visual areas, has been described in rodents (Laramée and Boire, 2014) and marmosets (Yu et al., 2020) and may reflect the brain size and computational complexity (Kondo et al., 2016). Rosa (2002) proposed that such fractured visual maps represent an evolutionary compromise, where local topographic coherence is maintained between adjacent cortical columns while allowing discontinuous field representations within areas to optimize specialized processing streams. However, partial representations of the visual field have also been observed in macaque V3 (Gattass et al., 1988), suggesting the brain size alone may not explain why fractured visual maps exist. Recent research has suggested that the reversals in the visual field do not necessarily denote area boundaries in the same way as previously anticipated (Rowley and Sedigh-Sarvestani, 2024). Rather, they emphasize how retinotopy can be nonlinear in nature. When these reversals are combined over a larger area, they form near-full coverage of the visual field (Zhuang et al., 2017; Yu et al., 2020; Rowley and Sedigh-Sarvestani, 2024). Similar nonlinear retinotopy has been observed in other species including tree shrews (Sedigh-Sarvestani et al., 2021), ferrets (Manger et al., 2002), and squirrels (Gould, 1984). It is possible that given their small body size, the reversals observed in marmoset PPC reflect a cohesive, nonlinear map of the visual field.

Large portions of the contralateral visual field could be represented across several hundred microns (Fig. 3). Furthermore, similar representations of the visual field at distinct recording locations were found (Fig. 4). Given that the PPC in the marmoset is thought to span several millimeters along the anterior posterior axis (Paxinos et al., 2012), our results suggest the representation of the visual field is repeated across the marmoset PPC. Consistent with this interpretation, human and macaque imaging studies have found multiple topographic maps in the PPC (Claeys et al., 2003; Medendorp et al., 2003; Bakola et al., 2006; Jack et al., 2007; Serences and Boynton, 2007; Patel et al., 2010; Arcaro et al., 2011), noting similar reversals in the orientation of the visual field on the boundaries between functional subregions. These subregions were observed to encode different motor functions including smooth and saccadic eye movements, reaching movements, and hand and grip movements (Andersen and Buneo, 2002) and may show differences in visual and cognitive behavior (Bakola et al., 2006; Patel et al., 2010). While representations other than preferred saccade direction were not tested for, the distinct and multiple representations of the visual field found in marmoset PPC (Figs. 3, 4) may reflect different areas of functional specialization within PPC.

The reasons why retinotopic organization decreases moving up the visual hierarchy remain unclear. One hypothesis is that decreased retinotopy and lateralization in higher-order visual areas allows for more complex, cognitive computations (Felleman and Van Essen, 1991; Jack et al., 2007; Patel et al., 2010). Another potential explanation for inconsistencies in reports of retinotopy in the PPC could be due to the differences in the nature of the task between these studies. When stimuli tended toward more passive viewing tasks, electrophysiological studies did not yield strong retinotopic maps in PPC (Blatt et al., 1990; Ben Hamed et al., 2001; Patel et al., 2010). However, when stimuli more actively engage cognitive functions like visual search, more complete topographic maps in some regions of the PPC can be observed (Patel et al., 2010). Furthermore, studies using oculomotor tasks noted that PPC favored topographic organization of saccade trajectories (Kagan et al., 2010; Savaki et al., 2010) that were responsive to both the contralateral and ipsilateral visual field (Platt and Glimcher, 1998; Claeys et al., 2003; Medendorp et al., 2003; Jack et al., 2007; Serences and Boynton, 2007). In this study, a saccade task was used, and a topographic pattern in marmoset PPC was found, where both the contralateral and ipsilateral parts of the visual field were represented, with a bias toward the contralateral field. Our results are consistent with those observed in human and macaque PPC (Schluppeck et al., 2006; Silver and Kastner, 2009; Patel et al., 2010; Arcaro et al., 2011).

The PPC is an area within the frontoparietal attentional network of primates that is thought to be an integrative hub, combining and transforming bottom–up visual information such as eye position, with top–down cognitive factors like attention. Neural activity within the PPC area LIP has been shown to be modulated by complex cognitive factors like visual attention (Goldberg et al., 1990; Colby and Goldberg, 1999; Bisley and Goldberg, 2010), reward (Platt and Glimcher, 1998; Sugrue et al., 2004), and decision-making (Gold and Shadlen, 2000; Hawellek et al., 2016; Wong et al., 2016). The PPC is directly and reciprocally connected to the frontal eye fields (FEF) and within the frontoparietal network (Collins et al., 2005; Reser et al., 2013; Ghahremani et al., 2017). In FEF, visually responsive neurons, which reflect retinal topography (Mohler et al., 1973; Schmolesky et al., 1998; Cavanaugh et al., 2012; Feizpour et al., 2021), are largely found laterally, while motor neurons are more medially (Sommer and Wurtz, 2006). Electrical stimulation of neurons in ventrolateral FEF evokes smaller amplitude foveal saccades; while in the dorsomedial regions of FEF, it evokes larger amplitude peripheral saccades (Bruce et al., 1985). Given the direct anatomical connections and coactivation of PPC with FEF in oculomotor control tasks (Barbas and Mesulam, 1981; Schall et al., 1995; Reser et al., 2013), subregions of PPC may have similar topographic maps of visual space. Accordingly, it is likely that the multiple representations of the visual field recorded in PPC in this study reflect subregions corresponding to elements such as neuron type (visual vs motor responsive) and saccade metric (i.e., direction, amplitude).

Overall, this study provides the first fine-scale evidence of a topographic organization of saccade direction preferences in marmoset PPC. Saccade direction preferences change systematically across cortical distance such that adjacent multiunits have smaller differences in preferred direction. Across larger cortical distances, this pattern of topographic organization in PPC is more inhomogeneous and fractured, reflecting multiple maps of the visual field. The results in this study provide insights into the complexity of computations that occur in PPC and suggest that circuits here are structured to mediate both local interactions in visual space and more complex interactions across regions of the PPC and the wider frontoparietal saccade network.

## Synthesis

Reviewing Editor: David Schoppik, New York University Grossman School of Medicine Decisions are customarily a result of the Reviewing Editor and the peer reviewers coming together and discussing their recommendations until a consensus is reached. When revisions are invited, a fact-based synthesis statement explaining their decision and outlining what is needed to prepare a revision will be listed below. The following reviewer(s) agreed to reveal their identity: NONE. Note: If this manuscript was transferred from JNeurosci and a decision was made to accept the manuscript without peer review, a brief statement to this effect will instead be what is listed below.

The revised manuscript addresses the reviewers' concerns and is the stronger for it.
